# Evaluation of the detection of Toll-like receptors (TLRs) in cancer development and progression in patients with colorectal cancer

**DOI:** 10.1371/journal.pone.0197327

**Published:** 2018-06-08

**Authors:** Ippokratis Messaritakis, Maria Stogiannitsi, Asimina Koulouridi, Maria Sfakianaki, Alexandra Voutsina, Afroditi Sotiriou, Elias Athanasakis, Evangelos Xynos, Dimitris Mavroudis, Maria Tzardi, John Souglakos

**Affiliations:** 1 Laboratory of Translational Oncology, Medical School, University of Crete, Heraklion, Crete, Greece; 2 Department of Internal Medicine B, Venizeleio Pananeio General Hospital, Heraklion, Crete, Greece; 3 Department of General Surgery, Heraklion University Hospital, Heraklion, Crete, Greece; 4 Creta Interclinic Hospital of Heraklion, Crete, Greece; 5 Department of Medical Oncology, University General Hospital of Heraklion, Crete, Greece; 6 Laboratory of Pathology, University General Hospital of Heraklion, Crete, Greece; Istituto di Ricovero e Cura a Carattere Scientifico Centro di Riferimento Oncologico della Basilicata, ITALY

## Abstract

**Background:**

Toll-like receptors (TLRs) play essential role in innate and acquired immunity, are expressed in various cell types, and are associated with altered susceptibility to many diseases, and cancers. The aim of this study was to investigate *TLR2* (-196 to-174*del*), *TLR4* (Asp299Gly and Thr399Ile) and *TLR9* (T1237C and T1486C) gene polymorphisms at risk of colorectal cancer (CRC) development and progression.

**Methods:**

Peripheral blood was obtained from 397 patients with adjuvant (stage II/III, n = 202) and metastatic (n = 195) CRC. Moreover, blood samples from 50 healthy volunteers and 40 patients with adenomatous polyps were also included as control groups. DNA from patients and controls was analyzed using PCR and PCR-RFLP for genotyping functional polymorphism within *TLR2*, *TLR4* and *TLR9* genotypes.

**Results:**

*TLR2–*196 to-174*del*/*del* genotype was detected in 76.6% of the patients and was significantly higher that the controls groups (*p*<0.001). *TLR4* Asp299Gly, *TLR4* Thr399Ile, *TLR9 T1237C* and *T1486C* homozygous genotypes were detected in 70.5%, 70.5%, 61.5% and 61.5% of the patients respectively, and were also significantly higher than that in the control groups (*p*<0.001). All polymorphisms detected were also significantly associated with the metastatic disease (*p*<0.001) leading to shorter overall survival (*p*<0.001); whereas, *TLR4* Asp299Gly and Thr399Ile polymorphisms were significantly associated with *KRAS* mutations.

**Conclusions:**

The detection of higher frequencies of the *TLR2*, *TLR4* and/or *TLR9* polymorphisms in CRC patients compared with the control groups highlight the role of these polymorphism in CRC development and cancer progression.

## Introduction

Colorectal cancer (CRC) represents the 9% of all malignancies and is considered as an important cause of death worldwide [1]. The development of CRC has been associated with genetic mutations, diet, inflammatory processes and the gut microflora, with the 95% of CRC cases been characterized as sporadic cancer [2]. The relationship between cancer and the microorganisms has been shown in various organs, with the most known example being the relation between *H*. *pylori* and gastric cancer [3].

Toll-like receptors (TLRs) play an essential role in both innate and acquired immunity and are expressed in various types of cells, including cancer cells [4–6]. Several polymorphisms within the TLR genes are associated with altered susceptibility to many diseases (infections, allergies, inflammatory diseases) as well as cancers [7] and TLR signaling plays a role in promoting malignant cell survival [8].

CRC development and progression have been correlated with *TLR2* and *TLR4* overexpression [9–10]. In fact, variant -196 to -174 chromosome 4q32 22-bp *insertion/deletion* (*Ins/Del*) polymorphisms in the promoter region, alters *TLR2* promoter activity and thus its level of expression [11]. Moreover, *TLR4* is expressed in CRC and promotes cancer cells to escape immune-surveillance by stimulating immunosuppressive agents and resistance to apoptosis [12]. Two common mutations, *Asp299Gly* and Thr399Ile, which occur in exon 4 of the human *TLR4* gene (A896G and C1196T, respectively), are located within the extracellular domain of the receptor [13], have been associated with increased risk to Crohn’s disease and ulcerative colitis [14–16]. Additionally, human *TLR9* occurs at 3p21.3, a region frequently deleted in human cancers [17]. Among the studied polymorphisms, variants T1237 and *T1486C* have been associated with the risks of multiple tumors; however, due to limited sample sizes, the reported results are inconsistent.

Herein, we hypothesized that the presence of *TLR2*, *TLR4* and *TLR9* variants affect gut homeostasis resulting in impairment of TLRs activation, thus leading to inflammation and CRC development and progression. Thus, we aimed to a) evaluate the expression of -196 to -174 *del* allele in the *TLR2* gene, the *Asp299Gly* and *Thr399Ile* polymorphisms in the *TLR4* gene and the T1237 and *T1486C* polymorphisms of the *TLR9* gene in patients with early CRC; b) compare their expression in patients with metastatic disease; c) determine whether patients carrying TLR alleles have an increased risk of recurrence and decreased overall survival and d) associate TLR polymorphisms with patients’ molecular profiling. These results are expected to show whether patients carrying TLR alleles have an increased risk of recurrence and decreased overall survival. Finally, the understanding of how TLRs enhance angiogenesis could help improve the development of anticancer agents and targeted therapies.

## Materials and methods

### Patients’ population

Since September 2003 to November 2013, 397 consecutive patients with newly diagnosed colon adenocarcinoma, treated at the Department of Medical Oncology, University Hospital of Heraklion were enrolled in the study. Disease status was coded, without the knowledge of the laboratory analysis. The study was approved by the Ethics Committee/Institutional review board of the University Hospital of Heraklion and signed informed consent has been obtained from all enrolled patients.

### Blood and tissue samples from control groups

Peripheral blood (15 ml in EDTA) was obtained from 50 healthy blood donors and was used as controls in the study. Moreover, formalin-fixed paraffin embedded (FFPE) tissues from 40 patients with colon adenomas, in the absence of CRC, were used as extra controls in the study.

### Genomic DNA extraction

Peripheral blood mononuclear cells (PBMC) from patients and healthy blood donors were obtained by Ficoll–Hypaque density gradient (*d* = 1,077 g/ml; Sigma-Aldrich, GmbH, Germany) centrifugation at 1,800 rpm for 30 min.

Representative formalin-fixed, paraffin-embedded (FFPE) primary tumor specimens were reviewed by an experienced pathologist in order to ensure the validity of the specimen and define the most appropriate area for microdissection. Malignant cells were procured using a piezoelectric micro dissector (Eppendorf, Germany) as previously described [18].

DNA extraction both from blood and tissue samples was performed using the MasterPure^™^ Complete DNA and RNA Purification Kit (Epicenter, Madison, Wisconsin, USA) following the manufacturer’s instructions. DNA was quantified using the NanoDrop ND-1000 v3.3 (ThermoFisher Scientific, Waltham, Massachusetts, USA) equipment and the samples were stored at -20°C until their use.

### TLR genotyping

Polymerase chain reaction (PCR) and restriction fragment length polymorphism (RFLP) was performed for genotyping of single nucleotide substitutions of the *TLR2*, *TLR4* and *TLR9* genes. Primers used for PCR amplification of the fragments are shown in S1 Table. In brief, determination of *TLR2*–196 to -174 *Ins*/*Del* polymorphism was performed by PCR as previously described [19]; while *TLR4* (*Asp299Gly* and Thr399Ile) and *TLR9* (T1237 and T1486C) genotyping were determined by PCR-RFLP. Allele types, single nucleotide polymorphism reference numbers and PCR conditions for all the analyzed polymorphisms are shown in S2 Table.

The amplified products of the *TLR4* (*Asp299Gly* and Thr399Ile) and *TLR9* (T1237 and T1486C) genes were digested using restriction enzymes; *Nco*I (ThermoFisher Scientific), *Hinf*I (New England BioLabs, United Kingdom), *BstN*I (New England BioLabs) and *Afl*II (New England BioLabs), respectively, according to manufacturer’s instructions. Briefly, 10 μl of each related PCR product was mixed with 1.5 μl of each restriction enzyme, 2 μl of 10× buffers and 7 μl H_2_O. After incubation at 37°C for 16 h, the restriction fragments were separated by electrophoresis on a 3% agarose gel, stained with Sybr Safe DNA Gel Stain (ThermoFisher Scientific), and visualized with the AlphaImager ultraviolet transilluminator (Alpha Innotech Corp., San Leandro, CA).

### Study design and statistics

This is a retrospective, single institution study, investigating the *TLR2*, *TLR4* and *TLR9* gene polymorphisms in PBMCs from patients with CRC before the initiation of adjuvant or first-line treatment. Disease-free survival (DFS) was calculated from the date of surgery to the date of disease recurrence, diagnosis of new colorectal primary or death from any cause, while progression-free survival (PFS) was calculated from the day of diagnosis to documented disease progression or death from any cause. Overall survival (OS) was calculated from the day of diagnosis to the date of death, from any cause. Laboratory analysis was carried out blindly to clinical data. Statistical analysis was based on contingency tables, including calculations of hazard rations (HR) and 95% CI. The two-tailed Fisher’s exact test was applied to determine whether distributions of categorized variables between the groups were significant. The association of risk factors with time-to-event endpoints was analyzed with the log rank test and the Kaplan–Meier method was used to plot the corresponding DFS, PFS and OS curves. The Hardy–Weinberg equilibrium was tested by comparing expected and observed genotype frequencies by chi-square test. Statistical significance was set at p = 0.05.

## Results

### Patients’ demographics and molecular characteristics

From 09/2013 to 11/2013, 397 newly diagnosed and histologically documented patients with CRC were enrolled in the study. The patients’ characteristics are listed in Table 1. The median age was 65 years, 246 (62.0%) patients were males, 202 (50.9%) was of stage IIA-IIIC, 279 (70.3%) had a colon/sigmoid tumor location, 372 (93.7%) had PS (ECOG) 0–1, and 205 (47.4%) had a high grade tumors. Moreover, 347 (87.8%) and 80 (20.4%) patients underwent surgery and radiotherapy, respectively; 230 (58.7%) patients received an adjuvant treatment, 64 (27.8%) of which were relapsed, whereas 223 (56.2%) patients received front-line treatment, 197 (88.3%) of which relapsed (Table 1 and S3 Table).

**Table 1:** Patients’ demographics and tumor’s characteristics.

	Frequency	%
**Age (range)**	65 (18–88)	
**<70**	260	65,5
**> = 70**	137	34,5
**Gender**		
**Male**	246	62,0
**Female**	151	38,0
**Stage**		
**IIA-IIIC**	202	50,9
**IV**	195	49,1
**Location**		
**Colon/Sigmoid**	279	70,3
**Rectum**	118	29,7
**PS (ECOG)**		
**0–1**	372	93,7
**> = 2**	25	6,3
**Radiotherapy**		
**Yes**	80	20,4
**No**	313	79,6
**Adjuvant Treatment**		
**Yes**	230	58,7
**No**	162	41,4
**First Line Treatment**		
**Yes**	223	56,2
**No**	174	46,8
**Grade**		
**High**	205	47,4
**Low**	228	52.6
**TLR2 196-to -174 del**		
**ins/del**	93	23,4
**del/del**	304	76,6
**TLR4 Asp299Gly (A/G)**		
**AA**	11	2,8
**AG**	106	26,7
**GG**	280	70,5
**TLR4 Thr399Ile (T/C)**		
**CC**	11	2,8
**CT**	106	26,7
**TT**	280	70,5
**TLR9 T1237C**		
**TC**	153	38,5
**CC**	244	61,5
**TLR9 T1486C**		
**TC**	153	38,5
**CC**	244	61,5
**KRAS**		
**Mutant**	104	42,4
**Wt**	141	57,6
**ND**	152	

### Analysis of the TLR2 gene (-196 to -174 del) polymorphisms

**Fig 1:**
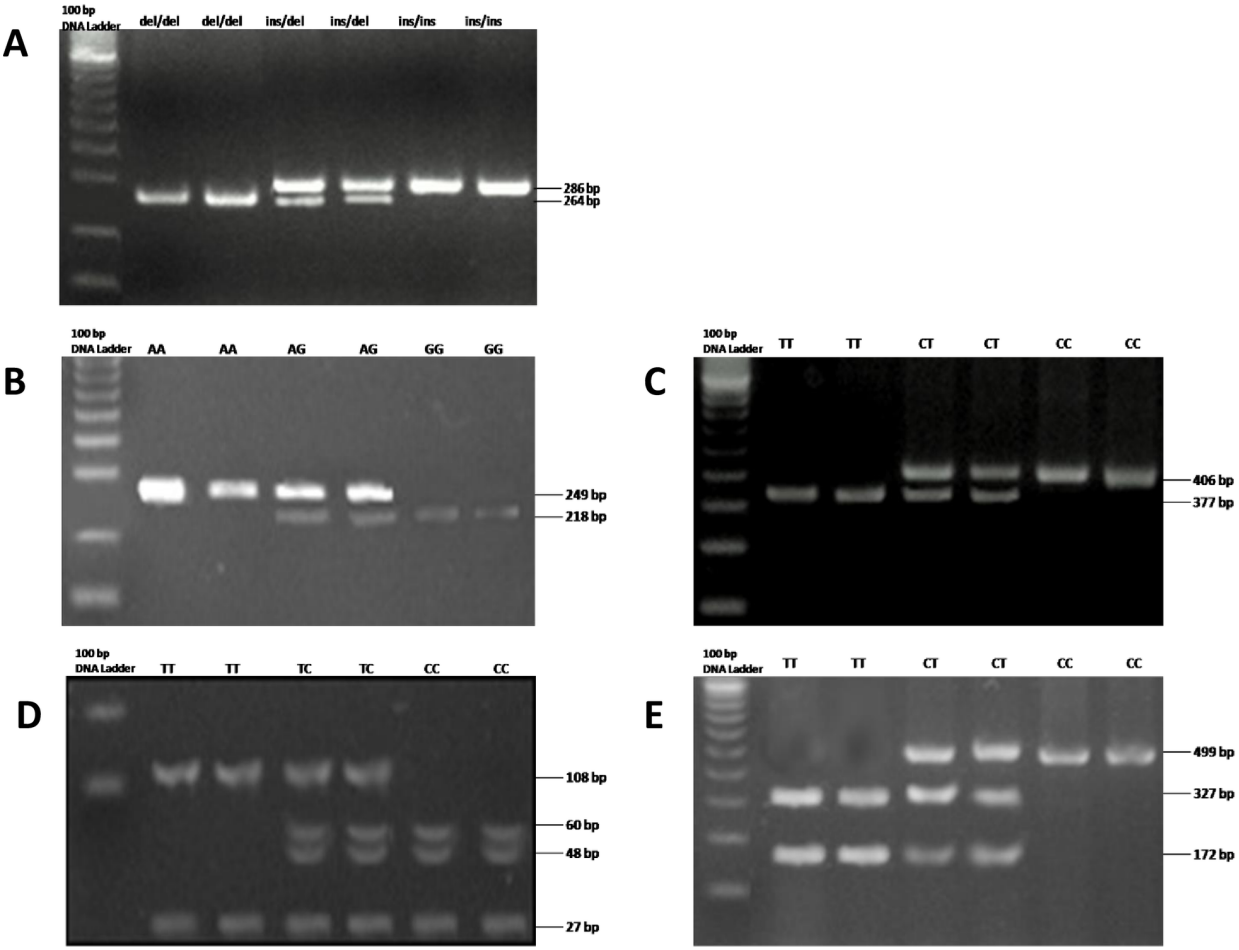
Representative agarose gels showing PCR-RFLP analysis of different single nucleotide polymorphisms of the a) TLR2–196 to 174 del, b) TLR4 Asp299Gly, c) TLR4 Thr399Ile, d) TLR9 T1276C and e)TLR4 T1486C, respectively.

Determination of the *TLR2* genotype was based on the presence of the specific band (Fig 1a). As indicated by the PCR gel electrophoresis, 93 (23.4%) patients presented heterozygous (*ins*/*del*) alleles, by simultaneously presenting the 286 bp 218 bp bands; whereas 304 (76.6%) patients presented the homozygous mutant (*del*/*del*) allele, by amplifying an 286 bp band (Table 1 and Fig 1a). Compared with the healthy donors and the patients with adenomatous polyps control groups, there was a significant association (*p*<0.001) in genotype and allelic frequency, since both control groups presented only the homozygous for the wild type (*ins*/*ins*) allele, by amplifying only the 286 bp band (Table 2, Fig 1a and S3 Table).

**Table 2:** Association of patients’ and control groups TLR gene polymorphisms.

Gene	SNP	Genotype	Patient No (%)	Healthy blood donors No (%)	Adenomatous polyps No (%)	*p* value
**TLR2**	**-196 to -174 bp**	**ins/ins**	0 (0,0)	50 (100,0)	40 (100,0)	<0.001
		**ins/del**	93 (23,4)	0 (0,0)	0 (0,0)	
		**del/del**	304 (76,6)	0 (0,0)	0 (0,0)	
**TLR4**	**Asp299Gly (A/G)**	**Wt**	11 (2,8)	50 (100,0)	36 (90,0)	<0.001
		**Hetero**	106 (26,7)	0 (0,0)	4 (10,0)	
		**Homo**	280 (70,5)	0 (0,0)	0 (0,0)	
	**Thr399Ile (T/C)**	**Wt**	11 (2,8)	50 (100,0)	36 (90,0)	<0.001
		**Hetero**	106 (26,7)	0 (0,0)	4 (10,0)	
		**Homo**	280 (70,5)	0 (0,0)	0 (0,0)	
**TLR9**	**T1237C**	**Wt**	0 (0,0)	26 (52,0)	10 (25,0)	<0.001
		**Hetero**	153 (38,5)	24 (48,0)	30 (75,0)	
		**Homo**	244 (61,5)	0 (0,0)	0 (0,0)	
	**T1486C**	**Wt**	0 (0,0)	26 (52,0)	10 (25,0)	<0.001
		**Hetero**	153 (38,5)	24 (48,0)	30 (75,0)	
		**Homo**	244 (61,5)	0 (0,0)	0 (0,0)	

### Analysis of the TLR4 gene (Asp299Gly and TLR4 Thr399Ile) polymorphisms

The expected amplification products of the *TLR4* gene *Asp299Gly* (A/G) and *Thr399Ile* (T/C) polymorphisms were 249 bp and 406 bp, respectively. The PCR products, for *Asp299Gly* (A/G) and *Thr399Ile* (T/C) polymorphisms were digested by *Nco*I and *Hinf*I enzymes, respectively. Following electrophoresis, 280 (70.5%), 106 (26.7%) and 11 (2.8%) patients presented the homozygous mutant, the heterozygous and the homozygous wild type genotype, for both polymorphisms (Table 1 and Fig 1b and 1c). Compared with the control groups, there was a significant association (*p*<0.001) in genotype and allelic frequency of both polymorphisms, since the healthy donors control group presented only the homozygous wild type genotype. Moreover, 36 (90%) and 4 (10%) adenomatous polyps control patients presented the homozygous wild type and the heterozygous polymorphisms, respectively, and this was observed both in the cases of *Asp299Gly* (A/G) and *Thr399Ile* (T/C) polymorphisms (Table 2, Fig 1b and 1c and S3 Table).

### Analysis of the TLR9 gene (T1237C and T1438C) polymorphisms

The expected amplification product of the *TLR9* gene *T1237C* polymorphism was 108 bp and 27 bp; whereas the length of the specific ampification band of the *T1486C* polymorphism was 499 bp. The PCR products, for *T1237C* and *T1486C* polymorphisms were digested by *Afl*II and *Bst*NI enzymes, respectively. Following electrophoresis, 244 (61.5%) and 153 (38.5%) patients presented the homozygous mutant and the heterozygous genotype, for both polymorphisms. Not one of the patients presented the homozygous wild type genotype. (Table 1 and Fig 1d and 1e). Compared with the control groups, there was a significant association (*p*<0.001) in genotype and allelic frequency of both polymorphisms, since 26 (52%) and 24 (48%) healthy donors presented only the homozygous wild type and the heterozygous genotype, respectively; whereas, 10 (25%) and 30 (75%) adenomatous polyps control patients presented the homozygous wild type and the heterozygous polymorphisms, respectively, and this was observed in both *T1237C* and *T1486C* polymorphisms (Table 2, Fig 1d and 1e and S3 Table).

### Association of TLR2, TLR4 and TLR9 variants and disease stage

The association between the TLR variants analyzed and the patients’ disease stage is presented in Table 3. The *TLR2* homozygous mutant (*del*/*del*) allele was more prevalent in stage IV patients whereas, the heterozygous (*ins*/*del*) allele was mostly seen in stage IIA-IIIC patients (95.9% vs 57.9% and 4.1% vs 42.1%, respectively; *p*<0.001) (Table 3). The *TLR4 Asp299Gly* (A/G) homozygous mutant genotype was more prevalent in stage IV patients, whereas the heterozygous and homozygous wild type genotype were mostly prevalent in stage IIA-IIIC patients (84.1% vs 57.1%, 13.8% vs 39.1% and 2.1% vs 3.5%, respectively; *p*<0.001); and this was also observed in the case of *Thr399Ile* (T/C) polymorphism (Table 3). Moreover, the *TLR9 T1237C* and *T1486C* homozygous mutant genotypes were also more prevalent in stage IV patients, whereas the heterozygous genotype was mostly met in stage IIA-IIIC patients (73.3% vs 50.0% and 26.7% vs 50.0%, respectively; *p*<0.001) (Table 3 and S3 Table).

**Table 3:** Association of TLR2, TLR4 and TLR9 variants and patients’ disease stage.

Gene	SNP	Genotype	IIA-IIIC	IV	*p*-value
**TLR2**	**-196 to -174 bp**	**ins/del**	85 (42,1%)	8 (4,1%)	<0,001
		**del/del**	117 (57,9%)	187 (95,9%)	
**TLR4**	**Asp299Gly** (A/G)	**WT**	7 (3,5%)	4 (2,1%)	<0,001
		**Hetero**	79 (39,1%)	27 (13,8%)	
		**Homo**	116 (57,4%)	164 (84,1%)	
	**Thr399Ile** (T/C)	**WT**	7 (3,5%)	4 (2,1%)	<0,001
		**Hetero**	79 (39,1%)	27 (13,8%)	
		**Homo**	116 (57,4%)	164 (84,1%)	
**TLR9**	**T1237C**	**Hetero**	101 (50,0%)	52 (26,7%)	<0,001
		**Homo**	101 (50,0%)	143 (73,3%)	
	**T1486C**	**Hetero**	101 (50,0%)	52 (26,7%)	<0,001
		**Homo**	101 (50,0%)	143 (73,3%)	

### Association of TLR2, TLR4 and TLR9 variants and KRAS status

Table 4 shows the association of the TLR variants according to the patients’ *KRAS* status. The *TLR2* homozygous mutant (*del*/*del*) allele was more prevalent in patients with a wild type *KRAS* status whereas the heterozygous (*ins*/*del*) allele was mostly seen in *KRAS* mutants (86.5% vs 77.9% and 13.5% vs 22.1%, respectively), although not significant (*p* = 0.076). *Asp299Gly* (A/G) and *Thr399Ile* (T/C) *TLR4* homozygous mutant genotype were more frequent in *KRAS* mutants, whereas heterozygous genotypes were more prevalent in *KRAS* wild type patients (80.8% vs 70.2% and 15.4% vs 28.4%, respectively; *p* = 0.028). On the contrary, both *TLR9 T1237C* and *T1486C* homozygous mutant genotypes were more prevalent in *KRAS* mutants, whereas the heterozygous genotypes were more frequent in *KRAS* wild type patients, although not significant (68.3% vs 58.2% and 31.7% vs 41.8%, respectively; *p* = 0.106) (Table 4 and S3 Table).

**Table 4:** Association of TLR2, TLR4 and TLR9 variants according to KRAS status.

			KRAS		
Gene	SNP	Genotype	Wt	Mutant	p value
**TLR2**	**-196 to -174 bp**	**ins/del**	19 (13,5%)	23 (22,1%)	0,076
		**del/del**	122 (86,5%)	81 (77,9%)	
**TLR4**	**Asp299Gly** (A/G)	**WT**	2 (1,4%)	4 (3,8%)	0,028
		**Hetero**	40 (28,4%)	16 (15,4%)	
		**Homo**	99 (70,2%)	84 (80,8%)	
	**Thr399Ile** (T/C)	**WT**	2 (1,4%)	4 (3,8%)	0,028
		**Hetero**	40 (28,4%)	16 (15,4%)	
		**Homo**	99 (70,2%)	84 (80,8%)	
**TLR9**	**T1237C**	**Hetero**	59 (41,8%)	33 (31,7%)	0,106
		**Homo**	82 (58,2%)	71 (68,3%)	
	**T1486C**	**Hetero**	59 (41,8%)	33 (31,7%)	0,106
		**Homo**	82 (58,2%)	71 (68,3%)	

### Association of TLR2, TLR4 and TLR9 variants and clinical outcome

**Fig 2:**
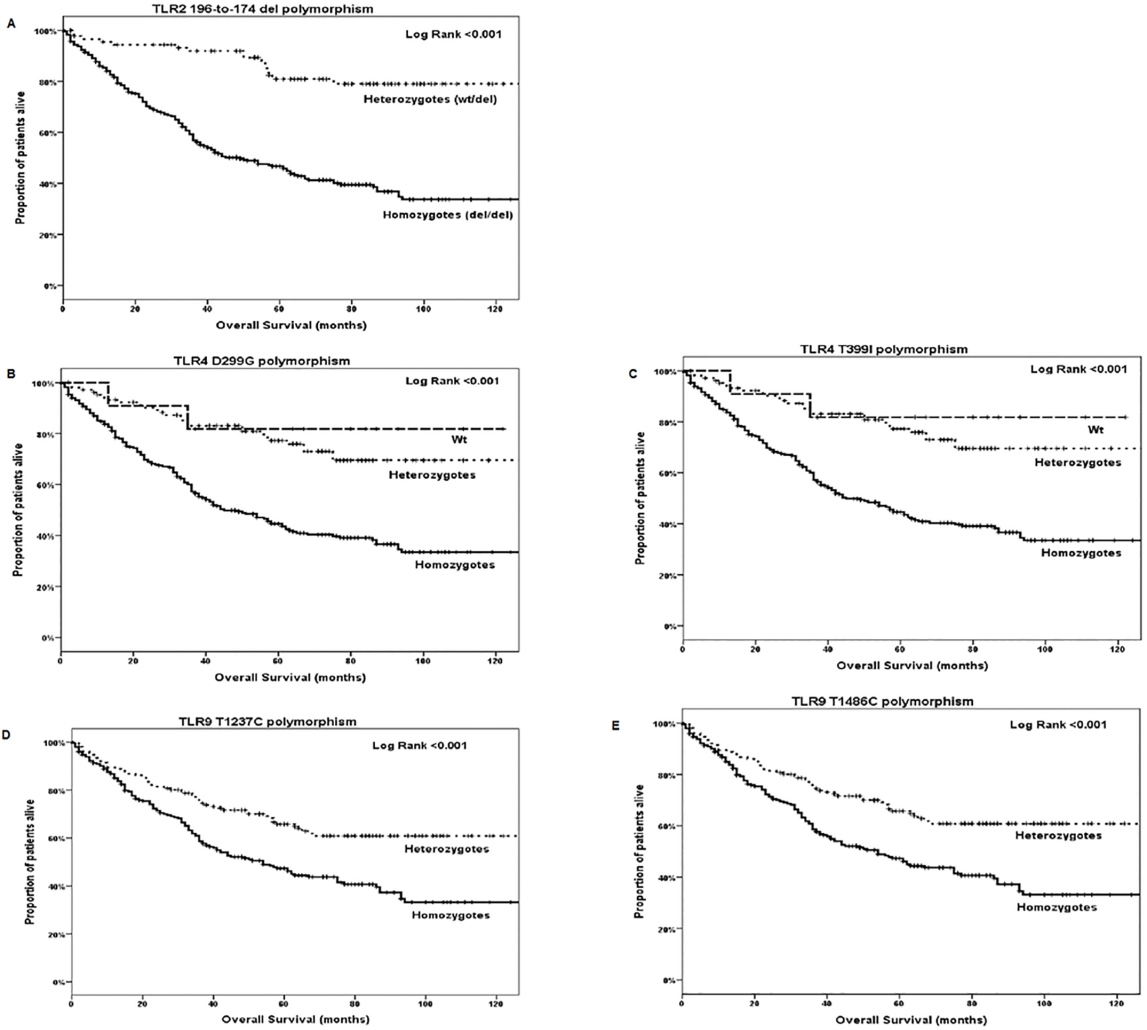
Overall survival according to the detection of a) TLR2–196 to -174 del, b) TLR4 Asp299Gly (A/G), c) TLR4 Thr399Ile (T/C), d) TLR9 T1276C and e) TLR4 T1486C, respectively.

A relapse of the adjuvant treatment was observed in 64 (27.8%) of the enrolled adjuvant patients, whereas 197 (49.7%) of the stage IV patients, relapsed following front line treatment (Table 1). The median DFS and OS for stage IIA-IIIC patients was 18 months (95% CI: 12.4–23.7) and 155 months (95% CI: 75.0–235.0), respectively. There was no difference of DFS or OS according to the presence of different TLR genotypes. The median PFS and OS for stage IV patients was 8 months (95% CI: 7.1–8.9) and 31 months (95% CI: 25.1–36.9), respectively. There was again no difference of PFS or OS according to the presence of different TLR genotypes. However, the median OS for all patients was 75 months (95% CI: 56.8–93.1) and this prevailed a significant shorter OS in patients with *TLR2* homozygous mutant (*del*/*del*) allele (*p*<0.001), *TLR4 Asp299Gly* (A/G) and *Thr399Ile* (T/C) homozygous mutant genotype (*p*<0.001 and *p*<0.001, respectively) or *TLR9 T1237C* and *T1486C* homozygous mutant genotype (*p*<0.001 and *p*<0.001, respectively) (Fig 2 and S3 Table).

## Discussion

The TLR pathway, through signaling by the myeloid differentiation primary response gene 88 (MyD88)-dependent pathway, increases the risk of colitis (or other inflammatory bowel diseases; IBDs)-associated CRC due to commensal gut microbiota [20–22]. In the present study, we aimed to evaluate the detection of *TLR2* (-196 to -174 *del*), *TLR4* (*Asp299Gly* and Thr399Ile) and *TLR9* (*T1237C* and T1438C) polymorphisms in CRC patients.

The *TLR2* gene is located on the chromosome 4 and the -196 to -174 *del* (a 22 bp nucleotide deletion) polymorphism alters the promoter activity of this gene. *TLR2 del*/*del* genotype is reported to show decreased transactivation of responsive promoters [11]. Previous studies investigated the likely association between this -196 to -174 *del* variant and nasopharyngeal cancer risk; the authors showed a significant association, concluding that the *del*/*del* genotype may represent a potential biomarker for nasopharyngeal cancer risk [23]. In another study conducted in Brazil, the authors showed that the frequency of heterozygous (*ins*/*del*) and homozygous mutant (*del*/*del*) alleles was significantly different between gastric cancer patients and healthy blood donors [24]; whereas others did not find any significant difference between gastric cancer patients and control groups for this variant [25–26]. An explanation to these contradictory results may be the enrolment of patients with distinct or ethnic differences among these studies. Nihon-Yanagi *et al*., suggest that *TLR2* activation may play a role in sporadic CRC [9]. However, its role in CRC is also ambiguous. Studies have shown no significant differences in CRC development between wild type and *TLR2* deficient mice [8, 27]; whereas, others have shown an increase in tumor growth and of IL-6, IL-17A and STAT3 levels in *TLR2*-deficient CRC mice, and a protective role of TLR against colitis-associated CRC [8, 28]. Our results are consistent with these studies since we observed higher frequencies of the *TLR2 del* allele in the CRC patients group compared with the control groups, highlighting the role of this polymorphism in colorectal carcinogenesis. Moreover, higher frequencies of the *TLR2 del* allele were detected in advanced CRC patients compared with the stage IIA-IIIC, emphasizing the role of this polymorphism in CRC progression and in patients’ overall survival.

Likewise, TLR4 has been known as indicative molecule for the detection of predisposition to cancer [9–10, 29–30]. The *TLR4* gene is located in the chromosome 9 and single nucleotide polymorphisms in exon 3 lead to *Asp299Gly* and *Thr399Ile* substitutions [24]. The increased TLR4 levels have been correlated with poor prognosis in patients with gastric, prostate and colorectal cancer, although the results are still controversial. In fact, one study showed no association with the risk of gastric cancer [31], while another study observed that both polymorphisms were associated with gastric cancer development [32], despite both studies were performed in the same ethnic group. Moreover, studies have demonstrated that high expression of TLR4/MyD88 is associated with liver metastases and is an independent predictor for poor prognosis, both in colitis-associated and in sporadic CRC cases [33]. Controversial results have been shown by Davoodi and Seow, suggesting that both *TLR4 Asp299Gly* and *Thr399Ile* alleles are not associated with CRC risk [34]. Other studies showed that the detection *TLR4 Asp299Gly* polymorphism had a 4-fold higher risk for development of prostate cancer, among a North Indian population [35]. However, the results from a meta-analysis suggest that the *TLR4 Asp299Gly* polymorphism had a protective effect in prostate cancer; whereas, both *TLR4 Asp299Gly* and *Thr399Ile* polymorphisms were associated with an elevated gastrointestinal cancer risk [36–37]. In another study, the authors present a clear evidence for an association between *TLR4* polymorphisms and CRC, suggesting that these polymorphisms could possibly serve as biomarkers for decision making in CRC treatment [38]. Our results are in accordance with other studies that associate the polymorphisms with patients’ outcome; in fact, our results suggest that the presence of both *Asp299Gly* and *Thr399Ile* polymorphisms were significantly associated with cancer development and progression, due to the higher frequency of these polymorphisms in CRC patients, and especially of those with metastatic disease. Moreover, the expression of either *Asp299Gly* or *Thr399Ile* homozygote mutant polymorphism had a great impact on the patients’ survival.

The *TLR9* gene is located on chromosome 3. Several polymorphisms have been identified, which two of them are located in the promoter; *T1237C* in exon 2 and *T1486C* in intron 1 [39]. Several studies have shown that binding of TLR9 to CD4 cells can enhance their survival and therefore could potentiate antitumor responses by prolonging T-cell activity [40]. Moreover, it has been reported that the enhanced longevity of TLR9-stimulated mouse T cells *in vitro* was dependent on NF-κB activation and was associated with up-regulation of the antiapoptotic protein Bcl-xL[41]. Several *TLR9* polymorphisms have been associated with susceptibility to inflammatory processes [42–43], thus highlighting the role of this gene in immune responses for gut homeostasis and the development of chronic inflammation. It has also been demonstrated that among others, *TLR9* expression increases in active ulcerative colitis patients, and that the mRNA levels positively correlate with the severity of intestinal inflammation as well as with inflammatory cytokines [44–45]. Regarding the connection between polymorphisms in *TLR9* genes and the risk of CRC, not much is known. It has been reported that oligodeoxynucleotides targeting *TLR9* oppositely modulate DNA repair genes in tumor versus immune cells and enhance the antitumor activity of DNA-damaging chemotherapy and radiation therapy in preclinical mouse models [46]. Moreover, it has been demonstrated that *TLR9* stimulates a series of immune responses, promotes angiogenesis and disease progression while decreasing patients’ survival [47–48]. In our study, both *TLR9 T1237C* and *T1486C* polymorphisms were more frequent in CRC patients compared to the control groups, and especially in stage IV patients, indicating their role in cancer development and progression.

A critical role in the resistance to treatment in patients with lung, pancreatic and colorectal cancer is played by continuous activation of the Ras/MAPK pathway because of mutations in codon 12 of the *KRAS* gene [49]. In our study, we also aimed to associate the frequency of *TLR2*, *TLR4* and *TLR9* polymorphisms in patients with different *KRAS* status. We found a significant association between both the *TLR4 Asp299Gly* and *Thr399Ile* polymorphisms with the *KRAS* mutant patients; however, no association was observed between any of the *TLR2* or *TLR9* polymorphisms and the *KRAS* status. It is to note that *KRAS* status was not determined for about 40% of the patients enrolled in the study, and these results have to be treated with caution.

In conclusion, our findings indicate a significant role of *TLR2*–196 to 174 *del*, *TLR4 Asp299Gly*, *TLR4* Thr399Ile, *TLR9* T1273C and *TLR9 T1486C* polymorphic variants with susceptibility to CRC development and disease progression, thus affecting patients’ survival. Due to the retrospective design, the lack of validation group and missing data regarding molecular subtypes of the enrolled tumors’ specimens, these results should be interpreted with caution and may serve mostly as hypothesis generated data for the rational design of future studies. Clearly, prospective studies are needed to better understand which microbial species are linked to cancer predisposition and which species are linked to positive outcomes. Moreover, further studies are necessary to the understanding of the way TLRs boost angiogenesis aiming the development of new anticancer agents and targeted treatments.

## Supporting information

S1 Table PCR primers designed to amplify fragments harbouring the TLR single nucleotide polymorphisms (SNPs). (DOCX)

S2 Table Allele types, single nucleotide polymorphism (SNP) reference numbers, and PCR conditions. (DOCX)

S3 Table Patients minimal underlying data. (XLSX)
