# Supplementary material for: Evaluation of the detection of Toll-like receptors (TLRs) in cancer development and progression in patients with colorectal cancer
Source: PLoS One. 2018 Jun 8;13(6):e0197327. doi: 10.1371/journal.pone.0197327 (PMC5993256; doi:10.1371/journal.pone.0197327)
Supplement: S1 Table — (DOCX) [file pone.0197327.s001.docx]

**Supplementary Table 1.** PCR primers designed to amplify fragments harbouring the TLR single nucleotide polymorphisms (SNPs)

| Gene | SNP | Primer | Sequence | Fragment size |
| --- | --- | --- | --- | --- |
| TLR2 | ***-196 to -174del*** | Forward | 5′-CACGGAGGCAGCGAGAAA-3′ | 286 bp uncleaved |
|  |  | Reverse | 3′-CTGGGCCGTGCAAAGAAG-5′ | 264 bp |
| TLR4 | ***Asp299Gly*** | Forward | 5′-GATTAGCATACTTAGACTACTACCTCCATG-3′ | 249 bp uncleaved |
|  |  | Reverse | 3′-GATCAACTTCTGAAAAAGCATTCCCAC-5′ | 218 bp, 31 bp |
|  | ***Thr399Ile*** | Forward | 5′-GGTTGCTGTTCTCAAAGTGATTTTGGGAGAA-3′ | 406 bp uncleaved |
|  |  | Reverse | 3′-ACCTGAAGACTGGAGAGTGAGTTAAATGCT-5′ | 377 bp, 29 bp |
| TLR9 | ***T1237*** | Forward | 5'-ATGGGAGCAGAGACATAATGGA-3' | 108 bp, 27 bp uncleaved |
|  |  | Reverse | 3'-CTGCTTGCAGTTGACTGTGT-5' | 60 bp, 48 bp, 27 bp |
|  | **T1486C** | Forward | 5′-TCCCAGCAGCAACAATTCATTA-3′ | 499 bp uncleaved |
|  |  | Reverse | 3′-CTGCTTGCAGTTGACTGTGT-5′ | 327 bp, 172 bp |
