# Supplementary material for: Evaluation of the detection of Toll-like receptors (TLRs) in cancer development and progression in patients with colorectal cancer
Source: PLoS One. 2018 Jun 8;13(6):e0197327. doi: 10.1371/journal.pone.0197327 (PMC5993256; doi:10.1371/journal.pone.0197327)
Supplement: S2 Table — (DOCX) [file pone.0197327.s002.docx]

**Supplementary Table 2. Allele types, single nucleotide polymorphism (SNP) reference numbers, and PCR conditions**

| SNP | Allele type | Ref. number | PCR conditions |
| --- | --- | --- | --- |
| -196 to -174 del | 22-bp deletion |  | Initial heating at 95°C for 5 min, followed by 35 cycles of denaturing (at 95°C for 30 s), annealing (at 60°C for 40 s), and chain extension (at 72°C for 40 s), followed by a final extension step at 72 °C for 7 min |
| Asp299Gly | A→G transition | rs4986790 | Initial heating at 95°C for 4 min, followed by 30 cycles of denaturing (at 95°C for 30 s), annealing (at 55°C for 30 s), and chain extension (at 72°C for 30 s), followed by a final extension step at 72 °C for 7 min |
| Thr399Ile | C → T transition | rs4986791 | Initial heating at 95°C for 4 min, followed by 30 cycles of denaturing (at 95°C for 30 s), annealing (at 55°C for 30 s), and chain extension (at 72°C for 30 s), followed by a final extension step at 72 °C for 7 min |
| T1237C | T→C transition | rs5743836 | Initial heating at 95°C for 4 min, followed by 40 cycles of denaturing (at 95°C for 30 s), annealing (at 61°C for 20 s), and chain extension (at 72°C for 18 s), followed by a final extension step at 72 °C for 5 min |
| T1486C | T→C transition | rs187084 | Denaturation at 94°C for 4 min, followed by addition of the polymerase, and then samples were exposed to 30 cycles of denaturing (at 95°C for 30 s), annealing (at 60°C for 20 s), and chain extension (at 72°C for 30 s), followed by a final extension step at 72°C for 5 min |
